# Emodin Inhibits Inflammation, Carcinogenesis, and Cancer Progression in the AOM/DSS Model of Colitis-Associated Intestinal Tumorigenesis

**DOI:** 10.3389/fonc.2020.564674

**Published:** 2021-01-08

**Authors:** Yunsha Zhang, Weiling Pu, Mélanie Bousquenaud, Sarah Cattin, Jelena Zaric, Li-kang Sun, Curzio Rüegg

**Affiliations:** ^1^ School of Integrative Medicine, Tianjin University of Traditional Chinese Medicine, Tianjin, China; ^2^ Pathology Unit, Department of Oncology, Microbiology and Immunology, Faculty of Science and Medicine, University of Fribourg, Fribourg, Switzerland; ^3^ Institute of Traditional Chinese Medicine, Tianjin University of Traditional Chinese Medicine, Tianjin, China

**Keywords:** emodin, colorectal cancer, Chinese medicine, tumor microenvironment, inflammation, T lymphocytes

## Abstract

Colorectal cancer (CRC) is one of the most common cancer worldwide. Chronic inflammation contributes to CRC development and progression. Emodin, is a natural anthraquinone derivative with anti-oxidant, anti-inflammatory, and anti-tumor activities. We used the AOM/DSS model of colitis-associated intestinal tumorigenesis to characterize the effect of Emodin on inflammation and tumorigenesis at weeks 3, 5, and 14 after initiation with AOM. At all three time points, Emodin (50 mg/kg) reduced inflammatory cell (i.e. CD11b^+^ and F4/80^+^) recruitment, cytokine (i.e. TNFα, IL1α/β, IL6, CCL2, CXCL5) and pro-inflammatory enzymes (i.e. COX-2, NOS2) expression in the tumor microenvironment, while promoting recruitment of CD3^+^ T lymphocytes at 14 weeks. Emodin decreased the incidence of premalignant lesions (adenoma) at week 3, the incidence of dysplastic lesions and carcinomas at week 5, and the incidence, size and the invasiveness of carcinomas at week 14. Emodin also reduced the acute clinical intestinal symptoms (i.e. bleeding and diarrhea) during DSS treatment. *In vitro*, Emodin inhibited the expression of pro-inflammatory mediators by LPS-stimulated RAW 264.7 macrophages, and reduced viability, adhesion, migration, and fibroblasts-induced invasion of SW620 and HCT116 colon cancer cells. In conclusion, this work demonstrates that Emodin suppresses carcinogenesis-associated intestinal inflammation and prevents AOM/DSS-induced intestinal tumorigenesis and progression. These results instigate further studies on Emodin as a natural agent for the prevention or treatment of colorectal cancer.

## Introduction

Colorectal cancer (CRC) is one of the most common cancer and one of the main leading causes of cancer-related death worldwide (1, 2). Chronic inflammation plays a critical role in CRC development and its inhibition has therapeutic benefits (3, 4). Patients with inflammatory bowel diseases (IBD), such as ulcerative colitis and Crohn’s disease, have a higher risk of developing CRC (5). In clinical and experimental studies, non-steroidal anti-inflammatory drugs (NSAIDs) were shown to protect average risk individuals, IBD patients and adenoma polyposis coli (APC) patients against CRC development (4, 6). In addition, emerging evidence indicates that NSAIDs, in particular aspirin, given as adjuvant therapy improve CRC overall survival, especially for patients with high PTGS2 (COX-2) expression (7, 8). Besides the beneficial effects of low-dose aspirin in preventing coronary events (9), the long-term use of NSAIDs, in particular COX-2 inhibitors, is associated with increased risk of severe gastrointestinal, renal and cardiovascular complications (10). This paradoxical situation should instigate the search for alternative, natural anti-inflammatory substances to prevent and treat CRC (11, 12).

Emodin (1,3,8-trihydroxy-6-methylanthraquinone), a natural anthraquinone derivative, is an active compound found in the roots and rhizomes of numerous Chinese medicinal herbs, such as *Rheum palmatum*, *Polygonum cuspidatum*, and *Cassia obtusifolia*, used as a traditional medicine in China and Japan (13). Emodin is also one of the active compounds of many natural purgatives and is used as a laxative (14). Modern pharmacology has demonstrated that Emodin has hypoglycemic, anti-oxidant, anti-tumor and anti-inflammatory properties, among other effects (14, 15). Emodin exerts anti-tumor effects by modulating multiple hallmarks of cancer, including cell growth (i.e. suppression of proliferation, promotion of apoptosis and altered cellular redox status), epithelial-mesenchymal transition (EMT), invasion and metastasis, and tumor angiogenesis (14). Multiple oncogenic signaling pathways and molecules modulated by Emodin have been identified, including NF-κB, HER-2, HIF-1α, AKT/mTOR, STAT3, Wnt, p38/p53/Puma, and VEGFR-2 (16–18).

Emodin exerts tumor suppressive effects in CRC through multiple mechanisms: i) induction of cancer cell apoptosis *via* endoplasmic reticulum stress-dependent events (19), p38/p53/Puma signaling (18), reactive oxygen species (ROS) production, Ca^2+^ release and caspase 9 activation (20), mitochondrial cytochrome c-release and disruption of the Bcl2/Bax balance (21); ii) inhibition of Wnt signaling (22) by downregulating the Wnt co-activator p300 and upregulating the Wnt repressor HBP1 (23); iii) inhibition of VEGFR-2 signaling (17); iv) suppression of urokinase secretion by normal, premalignant and malignant colonic epithelial cells (24). Shimpo *et al*., demonstrated *in vivo* that Emodin reduced the incidence of neoplastic lesions in APC ^Min/+^ mice treated with dextran sodium sulfate (DSS) by decreasing proliferation of intestinal epithelial cells (25).

Here, we further characterized the tumor preventive and suppressive effects of Emodin on CRC carcinogenesis and progression. To this end, we used the azoxymethane (AOM)/DSS model of colitis-associated colon tumorigenesis, based on the induction of DNA mutations by AOM (initiation) and intestinal inflammation induced by DSS (promotion) (26). It has been previously shown that in this model the pathological processes leading to cancer are similar to those in humans CRC pathogenesis, including mucosal inflammation, and epithelial hyperplasia and dysplasia (27).

## Materials and Methods

### Reagents and Antibodies

Crystal violet, paraformaldehyde, fibronectin, laminin, poly L-lysine, AOM, DSS, Lipopolysaccharide (LPS), Tween80, methylcellulose, MTT, and Griess reagent were obtained from Sigma-Aldrich. Matrigel and collagen I were from Corning. Emodin was purchased from Chengdu Herbpurify Company (China). Purity was 95–99% as assessed by HPLC. For immunohistochemical (IHC) staining, the following primary antibodies were used: anti-F4/80 (MF4800, Caltag) and anti-IκBα (Cat No.710128, Invitrogen), anti-Ki67 (ab16667, Abcam), anti-CD11b (ab133357, Abcam), anti-CD3 (ab5690, Abcam), anti-CD206 (ab64693, Abcam), anti-COX-2 (Cat No.610204, BD Transduction Laboratories), anti-CD31 (Neomarkers, RB-10333-P), and anti-Vimentin (Cat No.5741, Cell Signaling Technology). For flow cytometry analyses the following directly-labelled antibodies were used: anti-Ly6C-FITC (clone HK1.4, Biolegend), anti-Gr1-Pacific Blue (clone RB6-8C5, Biolegend), anti-Ly6G-APC (clone 1A8, Bio Legend), anti-CD11b APC-Cy7 (clone N418, eBiosciences, ThermoFischer Scientific), anti-CD4-FITC (clone RM4-5, Biolegend), anti-CD8-PE (clone 53-6.7, eBioscience).

### Animal Experiments

The animal experimental model was approved by the Cantonal Office in Fribourg (2014_27_FR) and experiments were performed according to Swiss regulations for animal experimentation. Male BALB/c mice were purchased from Envigo (France) and maintained in individually ventilates cages with free access to water and food under controlled temperature (22 ± 2°C) and humidity (50 ± 10%). The AOM/DSS colitis-associated intestinal carcinogenesis model was established in the laboratory as previously described (27). Mice were randomly assigned into experimental and control groups. AOM/DSS treatment was carried out as following (**Supplementary Figure S1**): mice were intraperitoneal injected with AOM (10 mg/kg body weight) at day 0, followed at day 7 by one cycle of DSS (2% w/v) dissolved in drinking water for 7 days. Emodin (50 mg/kg) or the vehicle solution (*dd*H_2_O with 0.2% Tween80 and 0.5% methylcellulose) were administrated by gavage, starting 2 days before DSS administration and continued for 2 weeks (endpoint at week 3 for the phase of acute colitis) or 4 weeks (endpoint at week 5 for the early stage of tumorigenesis or at week 14 for full tumorigenesis). Mice were weighed once a week and regularly scored for well-being and feces symptoms (normal feces, 0; diarrhea, 1; diarrhea and bleeding, 2).

At week 3, 5, and 14 after AOM injection, mice were sacrificed and the intestine was collected for histopathological examination and further biological and biochemical analyses. Blood was collected by terminal bleeding used for flow cytometry analysis.

### Histopathological Examination

Removed intestines were flushed with cold PBS, their length and weight measured, and were opened longitudinally for initial macroscopic examination. The pictures of the open intestines were taken by a binocular (Leica microsystems) and the tumor area was quantified by Adobe Photoshop CS6. Half of the intestine was fixed as “Swiss-rolls” in 4% paraformaldehyde overnight. Hematoxylin and eosin (H&E) staining were performed on paraffin-embedded section as previously reported (28). For IHC staining, paraffin-embedded slides were deparaffinized in xylene and rehydrated through graded ethanol steps. Slides were heated in different retrieval buffers, such as citrate (pH 6.00), proteinase K (10 min) or EDTA (pH 9.0), blocked with 2% BSA and incubated with primary antibodies overnight at 4°C. Primary antibodies were used as following: anti-F4/80 (1:25 dilutions), anti-COX-2 (1:50 dilutions = 5 µg/ml), and anti-CD31 (1:50 dilutions), anti-Ki67, anti-CD11b, anti-IκBα (1/100 dilutions = 5 µg/ml) and anti-vimentin (1:100 dilutions), anti-CD3 and anti-CD206 (1:200 dilutions). For most antibodies we were not able to obtain the actual concentration. After washing for three times, slides were incubated with biotinylated secondary antibodies (Dako EnVision anti-rabbit HRP, or anti-rat HRP, 1:500 dilution), followed by Vectastain ABC Kit (Vector Laboratories). DAB peroxidase substrate (Sigma-Aldrich) was used to reveal the signal from antibody-peroxidase complex. Finally, sections were counterstained with hematoxylin and mounted with neutral resins.

All the slides (H&E and IHC staining) were scanned with a NanoZoomer 2.0 HT (Hamamatsu) and images were extracted by NDP view 2 software. Histological parameters such as colitis, hyperplasia, adenoma, dysplasia and carcinoma were determined by a trained pathologist in a blinded fashion following the criteria described by Tanaka T and Washington MK (26, 29).

For large tumors, five randomly selected high-power fields (20× objective) per sample were chosen, while for tumor smaller than five fields across the whole tumor area was considered. The IHC stained cells (i.e. F4/80^+^, COX-2^+^, CD3^+^, and CD11b^+^, with brown cytoplasm and blue nucleus) were counted and the average of the five fields was taken as the number of positive cells in the sample. Similarly, five high-power fields of H&E stained sections were selected randomly for fibroblasts counts (i.e. number of fibroblasts per 100 total stromal cells). Fibroblast were identified based on the characteristic elongated nucleus morphology.

### Cell Culture and Tumor Spheroids Culture

The human colorectal carcinoma cell lines SW620 (ATCC^®^CCL-227), HCT116 (ATCC^®^CCL-247), and RAW 264.7 were from the American Type Culture Collection (ATCC; Rockville, MD, USA). Dermal fibroblasts were previously obtained from human skin as described (30). Cells were cultured in RPMI GlutaMAX™ (SW620) or in DMEM GlutaMAX™ (HCT116) or in high glucose DMEM (RAW264.7) supplemented with 10% fetal calf serum (FCS) and 1% penicillin/streptomycin. All cell culture reagents were purchased from Life Technologies.

Spheroids generation and culture and the assays of tumor cells invasion induced by fibroblasts in 2D and 3D conditions were performed as described previously (30). Briefly, for 2D conditions, cancer cell spheroids were placed on top of a confluent fibroblasts’ monolayer or on matrix coated wells. For 3D conditions, assays were performed according to the 3D-On-Top method with spheroids placed on a first dense Matrigel layer and covered with a second low-density Matrigel layer. Fibroblasts were added directly in the Matrigel. Cells were previously transduced to express DsRED-LiveACT (Fibroblasts) or GFP (SW620 and HCT116) (30). Growth factors reduced Matrigel was used (Corning). Emodin (dissolved in DMSO, final concentration from 10 to 40 μM), or solvent (0.1% DMSO at final concentration, v/v) only, were added in the medium of the treatment groups.

### Cancer Cell Growth, Adhesion, and Migration Assays

Cell number was estimated by MTT method as previously reported (31). The final concentration of Emodin was between 10 and 40 μM. Tumor cell adhesion assays were performed as previously described (32). Briefly, HCT116 cell and SW620 cell were pre-treated with Emodin (10, 20, and 40 μM) or solvent (0.1% DMSO) for 24 h, and then plated (4 × 10^4^ cells/well) in a 96-well plate pre-coated with collagen I (10 μg/ml), fibronectin (6 μg/ml), or BSA (2%) and incubated for 3 h at 37°C. The attached cells were stained by crystal violet and quantified by OD reading at 620 nm. The migration ability of cancer cells was evaluated by the wound healing assay. Briefly, cells were seeded on 96-well plate and grown to 90% confluence. The precise wounds were created with Wound Maker™ and cellular debris removed with a PBS wash. The wound widths and the images of each well were recorded every one for 24 h with the Incucyte software.

### LPS-Induced RAW 264.7 Activation

RAW 264.7 cells (2.5 × 10^5^ cells/ml) were seeded in 24- or 96-well plate, and treated with 0.5 μg/ml LPS with or without Emodin at various concentrations (10, 20, 40, 60 μM) for 24 h. Cell viability was assessed by MTT assay. LPS-induced NO production in cell culture supernatant was measured by the Griess reaction. TNFα secreted from LPS-treated cells were determined by ELISA (R&D Systems) according to the manufacturer’s instructions.

### Flow Cytometry Analysis

Peripheral blood leukocytes were analyzed by flow cytometry following standard protocols. Briefly, red blood cells were lysed with BD FACS lysing solution (BD Biosciences), and then leukocytes were washed and stained with fluorescent-labelled antibodies following recommended dilutions. The antibodies used were the following: anti-Ly6C-FITC (1:100 dilution), anti-Gr1-Pacific Blue (1:200 dilution), anti-Ly6G-APC (1:100 dilution), anti-CD11b-APC-Cy7 (1:50 dilution), anti-CD4-FITC (1:100 dilution), anti-CD8-PE (1:100 dilution). Labeled cells were analyzed on a BD FACSCalibur. Data were analyzed using FlowJo software.

### Real-Time PCR

Total mRNA was extracted using RNeasy Mini kit (Qiagen) and was reverse transcription to DNA using Superscript First Strand Synthesis Kit (Invitrogen, Life Technology). PCR were performed using Real-Time PCR cycler (Bio-Rad) by SYBR Green PCR reagent (Kapa). The primer pairs used are listed in **Table 1**.

**Table 1:** Primers used for RT-PCR.

Gene name	Gene primer sequence	
	Forward 5’-3’	Reverse 5’-3’
*GAPDH*	GGACCTGACCTGCCGTCTAG	CCACCACCCTGTTGCTGTAG
*m36B4*	GTGTGTCTGCAGATCGGGTAC	CAGATGGATCAGGAAG
*TBP*	CCCTTGTACCCTTCACCAATGAC	TCACGGTAGATACAATATTTTGAAGCTG
*TNFα*	CCCCAAAGGGATGAGAAGTT	GGTCTGGGCCATAGAACTGA
*NOS2*	TGGTGGTGACAAGCACATTT	AAGGCCAAACACAGCATACC
*IL1α*	CCAGAAGAAAATGAGGTCGG	AGCGCTCAAGGAGAAGACC
*IL1β*	GGTCAAAGGTTTGGAAGCAG	TGTGAAATGCCACCTTTT GA
*COX-2*	GTATCAGAACCGCATTGCCTC	CGGCTTCCAGTATTGAGGAGAACAGAT
*CCL3*	ACCATGACACTCTGCAACCA	TCAGGCATTCAGTTCCAGGT
*CCL5*	ACCATGAAGATCTCTGCAGC	TGAACCCACTTCTTCTCTGG
*CXCL5*	GTTCCATCTCGCCATTCATG	GCGGCTATGACTGAGGAAGG
*CCL2*	CACTCACCTGCTGCTACTCA	GCTTGGTGACAAAAACTACAGC
*IL6*	TGATGCACTTGCAGAAAACA	ACCAGAGGAAATTTTCAATAGGC

### Statistical Analysis

The data were expressed as the mean ± SEM and analyzed by GraphPad Prism 6.0. D’Agostino-Pearson omnibus normality test was used to assess data distribution. Samples from two groups were analyzed using unpaired Student’s *t*-test or Mann-Whitney test in case groups would not pass normality test. When comparing more than two variables, one-way or two-way ANOVA were performed. Correlation analysis was tested using Pearson’s correlation or Spearman correlation coefficient. *p <*0.05 was considered statistically significant.

## Results

### Emodin Treatment Does Not Cause Toxicity in AOM/DSS Treated Mice

To monitor for possible toxic effects of Emodin treatment in the AOM/DSS model we measured body weight weekly during the first 5 weeks of treatment, and liver and kidney weight, two organs sensitive to systemic toxicity, at the end of week 5. AOM/DSS treatment (group AOM/DSS) caused a decrease in body weight compared to vehicle-only control groups (group WT), while there was no significant difference between the body weight of mice treated with AOM/DSS vs AOM/DSS/Emodin (group Emodin) (**Supplementary Figure S2**). In addition, there was no significant difference in liver weight index (mean value about 6% of body weight) and kidney weight index (mean value about 2% of body weight) of all groups, indicating that Emodin did not produce obvious toxicity at the tested dose (50 mg/kg).

### Emodin Inhibits AOM/DSS-Induced Colitis-Associated Tumorigenesis

**Figure 1:**
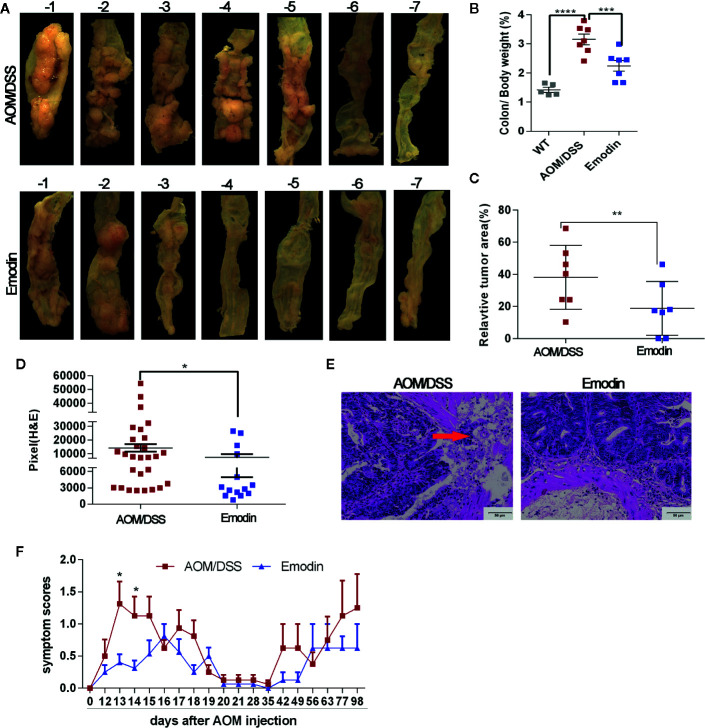
Emodin inhibits AOM/DSS-induced colitis associated tumorigenesis. **(A)** Representative macroscopic views of the tumor area of the colon at week 14 after tumor initiation. **(B)** Colon index (colon weight/body weight expressed in %) of tumor free mice (WT), AOM/DSS-treated mice (AOM/DSS) and AOM/DSS/Emodin-treated mice (Emodin). **(C)** Relative tumor surface area of group AOM/DSS and group Emodin evaluated by macroscopic analysis. **(D)** Relative microscopic tumor area of group AOM/DSS or group Emodin evaluated by microscopic analyses of H&E stained tumor sections. **(E)** Representative H&E stained sections of tumors showing strong tumor invasion in the AOM/DSS group while no invasion in the Emodin group (20× objective field). Arrow indicates invasive tumor area. **(F)** Scoring for feces at different time points after treatment initiation: normal 0, diarrhea 1, bleeding 2. WT, tumor free mice; AOM/DSS, AOM/DSS-treated mice; Emodin, AOM/DSS/Emodin-treated mice. WT, n = 5; AOM/DSS and Emodin, n = 7/group for **(A–E)**, n = 15/group for **(F)**. *p < 0.05, **p < 0.01, ***p < 0.001, ****p < 0.0001. Scale bar are shown in the panels.

At week 14 after initiation of tumorigenesis, macroscopically visible, nodular tumors were observed in the middle and distal colon of mice in the AOM/DSS group. Macroscopic and histological analysis showed that Emodin treatment decreased the incidence (**Table 2**) and the size of intestinal tumors (**Figure 1A** and **Supplementary Figure S3**). The colon index (colon/body weight in percent) **(** **Figure 1B**) and the relative macroscopic tumor area (**Figure 1C**) were markedly decreased in the Emodin treated group compared with vehicle only treatment. To evaluate tumor burden histologically, we measured the area of the tumor lesions on histological H&E sections, and confirmed that tumors in AOM/DSS/Emodin group (group Emodin) were smaller compared to tumors of AOM/DSS treated mice (**Figure 1D** and **Table 2**). Histological H&E analysis revealed that in the AOM/DSS group 5/7 mice (71%) had invasive tumors, while in the Emodin group (AOM/DSS/Emodin treated) only 1/7 of the mice (14%) had invasive tumors (**Figure 1E** and **Table 2**). Diarrhea and blood in feces are important clinical parameters to score the severity of AOM/DSS induced intestinal inflammation and tumorigenesis (27). Scoring for diarrhea and bleeding revealed that Emodin treatment decreased these symptoms immediately after the end of DSS treatment (**Figure 1F**).

**Table 2:** Incidence of colon neoplasms at 14 weeks after tumor initiation with AOM.

Group	No. of mice examined	No. of mice with carcinomas			No. of mice with invasive carcinomas
		Total	No. of large carcinomas	No. of small carcinomas	
AOM/DSS	7	7(100%)	5(71.4%)	7(100%)	5(71.4%)
		^$^5(3,7)	2(2,3)	3(1,4)
Emodin	7	5(71.4%)	2(40%)	5(100%)	1(14%)
		3(1.5,5)	2(2, 2)	3(1.5,3)

^$^Median (25th and 75th percentile). AOM/DSS, AOM/DSS treated mice; Emodin, AOM/DSS/Emodin treated mice.

Taken together, these results indicate that Emodin treatment suppresses tumor incidence, growth and invasion in AOM/DSS-treated mice, reduced bleeding and diarrhea, without clinical evidence of toxicity.

### Emodin Attenuates Inflammation in the Tumor Microenvironment

**Figure 2:**
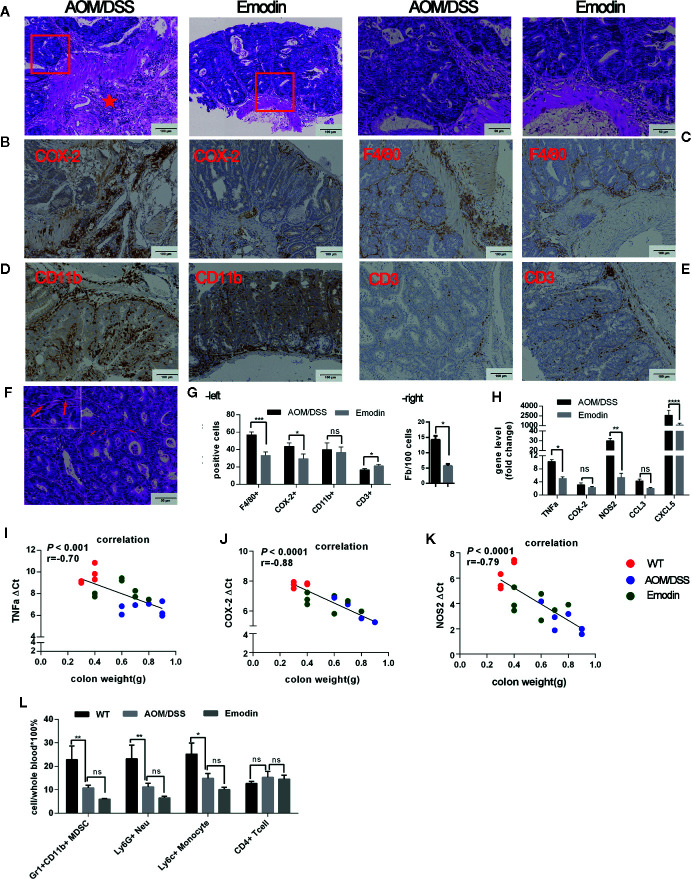
Emodin attenuates inflammation in the tumor microenvironment. **(A)** Representative images of H&E stained tumor sections at week 14 after tumor initiation showing infiltration of inflammatory cells. Star show the area with dense infiltration by inflammatory cells. **(B–E)** Immunohistochemistry staining for **(B)** COX-2, **(C)** F4/80, **(D)** CD11b, and **(E)** CD3 expression in the tumor microenvironment. **(F)** Fibroblasts in tumor stroma, identified by H&E staining. Arrows show the elongated nucleus of a fibroblast. **(G)** Quantification of inflammatory cells (-left) and fibroblasts (-right) in the tumor microenvironment. **(H)** Relative expression levels of mRNA for TNFα, COX-2, NOS2, CCL3, CXCL5 measured by RT-PCR. Expression (fold change) is compared to the expression in tumor free mice (WT). **(I–K)** Linear regression analysis of colon tissue weight and **(I)** TNFα, **(J)** COX-2, and **(K)** NOS2 gene expression levels analyzed by Pearson’s or Spearman correlation. Gene expression levels are presented as ΔCt, where high ΔCt means lower expression. **(L)** Relative frequency of Gr1^+^CD11b^+^, Ly6G^+^, Ly6C^+^, and CD4^+^ T cells in the peripheral blood measured by flow cytometry. WT, tumor free mice; AOM/DSS, AOM/DSS treated mice; Emodin, AOM/DSS/Emodin treated mice. WT, n = 5; AOM/DSS and Emodin, n = 7/group. *p < 0.05, **p < 0.01, ***p < 0.001, ****p < 0.0001, ns, not significant. Scale bar are shown in the panels.

Next, we evaluated the environment surrounding the tumor tissue in particular the inflammatory response at week 14 after tumor initiation. The histological analyses of the tissues around the tumor (H&E staining) demonstrated a striking recruitment of inflammatory cells in the submucosa of the colon in the AOM/DSS group compared to the AOM/DSS/Emodin group. Mice treated with Emodin had less aggressive tumors, and the submucosa surrounding the tumor tissue was less infiltrated with inflammatory cells (**Figure 2A**). To identify specific cell populations of the infiltrates, we performed IHC staining for selected molecules: CD3 for T cells; CD11b for myelomonocytic cells; CD31 for endothelial cells; F4/80 for macrophages; COX-2 for COX-2 expressing cells. IHC results showed that in the tumor microenvironment of Emodin-treated mice there were fewer COX-2 positive cells (**Figure 2B**), fewer F4/80^+^ macrophages (**Figure 2C**), and fewer CD11b^+^ cells (**Figure 2D**), yet slightly more CD3^+^ T cells (**Figure 2E**) compared to AOM/DSS treated mice. Visual differences were confirmed by quantification (**Figure 2G**, left panel). Since cancer associated fibroblasts can contribute to the occurrence and progression of colorectal cancer (33), we enumerated the fibroblasts present in the tumor stroma. Fibroblasts were identified on H&E sections based on their typical elongated cellular and nuclear morphology (**Figure 2F**). Indeed, Emodin reduced the presence of fibroblasts in the tumor environment (**Figure 2G**, right panel). Further, we monitored the expression of inflammation-associated genes by Real Time-PCR. The mRNAs level of transcripts for pro-inflammatory mediators such as TNFα, NOS2, CCL3, CXCL5 were significantly reduced in the Emodin group compared to the AOM/DSS group, while COX-2 mRNA expression was not significantly altered (**Figure 2H**). Furthermore, we observed a positive correlation between distal colon weight (as surrogate of tumor burden) and the mRNA levels of TNFα, NOS2, and COX-2 (**Figure 2I-2K**). This suggested that high expression of inflammation-related genes in the colon tissue correlated with increased tumor growth (gene level is presented as ΔCt: high ΔCt means lower expression).

To obtain evidence whether Emodin has systemic effects on inflammatory cells, we analyzed the phenotypes of leukocytes in the peripheral blood by flow cytometry. The percentage of Gr1^+^CD11b^+^ leukocytes (also referred to myeloid-derived suppressor cells – MDSCs) was significantly lower in group AOM/DSS relative to tumor free mice. Emodin treatment further reduced their frequency, but the difference with the AOM/DSS groups was not significant (**Figure 2L**). Consistently, changes in the frequency of Ly6G^+^ (neutrophils/granulocytic MDSCs) and Ly6C^+^ (monocytes/monocytic MDSC) paralleled changes in Gr1^+^CD11b^+^ cells. In contrast, no changes in the frequency of peripheral CD4^+^ T cell were observed (**Figure 2L**).

Taken together, these results indicate that Emodin treatment suppresses the recruitment of myeloid inflammatory cells (i.e. granulocytes and monocyte) and reduces the number of cancer associated fibroblasts, while enhancing the recruitment of T lymphocytes in the tumor microenvironment. Emodin also reduces expression of transcripts for inflammatory cytokines and decreases the frequency of granulocytes and monocytes in the peripheral blood.

### Emodin Suppresses Early Stage of Tumorigenesis and Associated Inflammation

**Figure 3:**
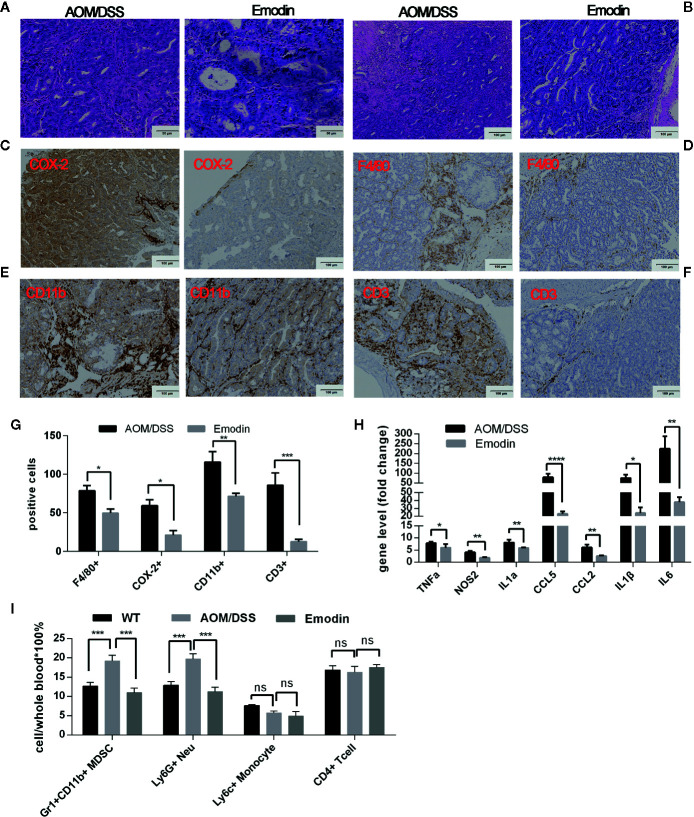
Emodin suppresses colon inflammation at the early stage of tumorigenesis (Week 5 after tumor initiation). **(A)** Representative histological images of tumor sections stained by H&E at week 5 after tumor initiation (20× objective field). Carcinoma are present in the AOM/DSS group; adenoma are present in the Emodin group. **(B)** Representative images of H&E sections from the same tumors showing infiltration by inflammatory cells. **(C–F)** Immunohistochemistry staining for **(C)** COX-2, **(D)** F4/80, **(E)** CD11b, and **(F)** CD3 in the tumor microenvironment. **(G)** Quantification of inflammatory cells in panels **(C–F)**. **(H)** Relative mRNA expression levels for TNFα, COX-2, IL1α, CXCL5, CCL2, IL1β, and IL6 measured by RT-PCR. Expression (fold change) is compared to expression in tumor free mice (WT). **(I)** Relative frequency of Gr1^+^CD11b^+^, Ly6G^+^, Ly6C^+^, and CD4^+^ T cells in the peripheral blood measured by flow cytometry. WT, tumor free mice; AOM/DSS, AOM/DSS treated mice; Emodin, AOM/DSS/Emodin treated mice. WT, n = 5; AOM/DSS and Emodin, n=8/group. *p < 0.05, **p < 0.01, ***p < 0.001, ****p < 0.0001, ns, not significant. Scale bar are shown in the panels.

As the inflammatory microenvironment plays an important role in tumor progression, we then investigated whether Emodin modulated intestinal inflammation already at earlier stages of tumorigenesis. To this end, we analyzed lesions at week 5 after tumor initiation with AOM and at the end of DSS treatment (week 4). The grade of colitis and the percentage of mice with either adenoma or carcinoma were determined by a gastrointestinal pathologist in a blinded manner. Emodin treatment significantly decreased the incidence of neoplasms (**Table 3**) and their histological grade. The most frequent lesions in the Emodin group were low grade dysplastic adenoma, while in the AOM/DSS group the most frequent lesions were high-grade dysplastic adenoma or carcinoma (**Figure 3A**). Strikingly, Emodin also reduced inflammatory cell recruitment and epithelial erosions compared to the AOM/DSS treatment group (**Figure 3B**).

**Table 3:** Incidence of colon inflammation and neoplasms at 5 weeks after tumor initiation with AOM.

Group	No. of mice examined	No. of mice with colitis		No. of mice with neoplasms		
		Grade1	Grade2	Total	adenoma	Carcinoma
AOM/DSS	8	2(2.5%)	4(50%)	7(87.5%)	2(25%)	5(62.5%)
					^$^1(1,2)	2(1.5,3)
Emodin	8	0(62.5%)	0(0.0%)	3(37.5%)	3(37.5%)	0(0.0%)
					1(1,2)	

^$^Median (25^th^ and75^th^ percentile). AOM/DSS, AOM/DSS treated mice; Emodin, AOM/DSS/Emodin treated mice.

We then performed IHC staining of cellular markers and of COX-2, a critical driver of colorectal carcinogenesis (34). COX-2 staining showed that in addition to positive expression in inflammatory cells in the group AOM/DSS, COX-2 was also strongly expressed in epithelial tumor regions, while in group Emodin (Emodin treatment), most of the tumor epithelial cells showed little to no expression of COX-2 (**Figure 3C**). F4/80 staining (**Figure 3D**) showed that the presence of F4/80^+^ macrophages within the tumor was low in both AOM/DSS and Emodin groups. F4/80 positive macrophage were more present in the tumor periphery and in the muscle layer of the submucosa, and Emodin significantly reduced their presence (**Figure 3D**). CD11b staining showed that Emodin reduced the infiltration by CD11b^+^ myelomonocytic cells relative to AOM/DSS treatment group (**Figure 3E**). CD3 staining revealed that Emodin strongly decreased T cells infiltration in the tumor (**Figure 3F**), in contrast to what we observed at week 14. The quantification of the IHC findings is given in **Figure 3G**. We also monitored the expression of inflammatory transcripts by RT-PCR. Consistent with the cellular findings, the expression of transcripts for the inflammatory mediators TNFα, NOS2, IL1α, IL1β, IL6 and CCL5 were significantly inhibited by Emodin (**Figure 3H**). In the peripheral blood we observed an increase in the fraction of Gr1^+^CD11b^+^ myeloid cells and Ly6G^+^ granulocytic MDSCs in the AOM/DSS group relative to tumor free mice. Emodin treatment reversed this effect (**Figure 3I**). Ly6C^+^ (monocytic MDSC) and CD4^+^ T cells were unchanged.

Taken together, these results indicate that Emodin reduces inflammatory cell recruitment, inflammatory cytokines expression, epithelial hyperplasia/dysplasia and carcinoma formation at early stage of tumorigenesis induced by AOM/DSS treatment.

### Emodin Suppresses Inflammation Elicited by DSS

**Figure 4:**
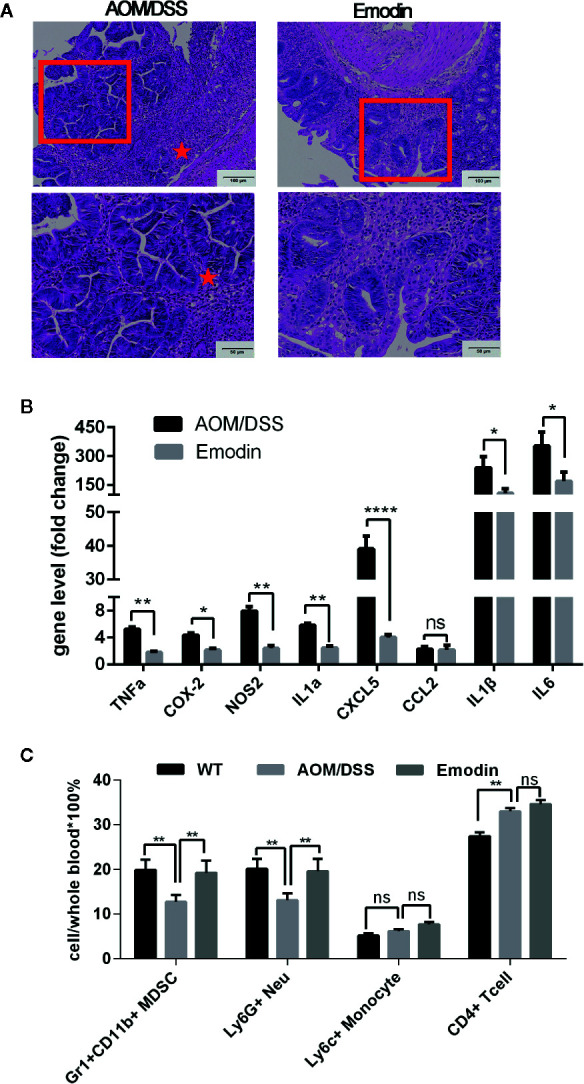
Emodin suppresses the acute inflammatory response. **(A)** Representative histological images of the intestine at week 3 after tumor initiation with AOM and 1 week after the end of tumor promotion with DSS (H&E staining). Erosions, hyperplastic and dysplastic epithelium, and massive inflammatory cell infiltrates are well visible in AOM/DSS-treated mice, while in AOM/DSS/Emodin-treated mice there rare erosions, some hyperplastic but no dysplastic epithelium and reduced inflammatory cell infiltrates. Stars indicate the area with severe erosion and dense inflammatory cell infiltrates. **(B)** Relative expression levels of mRNA for TNFα, COX-2, NOS2, IL1α, CXCL5, CCL2, IL1β, and IL6 measured by RT-PCR. Expression (fold change) is compared to expression in tumor free mice (WT). **(C)** Relative frequency of Gr1^+^CD11b^+^, Ly6G^+^, Ly6C^+^, and CD4^+^ T cells in the peripheral blood measured by flow cytometry. AOM/DSS, AOM/DSS treated mice; Emodin, AOM/DSS/Emodin treated mice. WT, n = 5; AOM/DSS, n = 10; Emodin, n = 9. *p < 0.05, **p < 0.01, ****p < 0.0001. ns, not significant. Scale bar are shown in the panels.

Stimulated by these results, we examined whether Emodin modulated inflammation during the acute inflammatory phase induced by DSS (tumor promotion). To this end, we examined Emodin effects on acute inflammation at one week after the end of DSS administration. Clinically, Emodin treatment decreased clinically symptoms associated with acute, severe intestinal inflammation (i.e. diarrhea and bleeding) (**Figure 1F**). Consistent with these clinical symptoms, histological analysis showed that Emodin reduced erosion, inflammatory cell recruitment and epithelial hyperplasia/dysplasia (**Figure 4A**). Quantification of the grade of colitis and epithelial dysplasia confirmed that the grades of inflammation and dysplasia in the AOM/DSS/Emodin group were lower compared to the AOM/DSS group (**Table 4**). Furthermore, treatment with Emodin caused a marked decrease in mRNA expression levels of the inflammatory mediators TNFα, COX-2, IL1a/β, NOS2, CXCL5, CCL2, and IL6 (**Figure 4B**). Flow cytometry analysis of circulating leucocytes revealed that the percentages of Gr1^+^CD11b^+^ and Ly6G^+^ positive neutrophils, but not Ly6C^+^ positive monocytes, were significantly decreased in the AOM/DSS group relative to tumor free-mice (WT). Strikingly, Emodin treatment reversed the AOM/DSS effect (**Figure 4C**). In contrast, the frequency of circulating CD4^+^ T cells increased in both AOM/DSS and Emodin groups, relative to tumor free-mice (WT) (**Figure 4C**).

**Table 4:** Incidence of colon inflammation and dysplasia at 3weeks after tumor initiation with AOM.

Group	No. of mice examined	No. of mice with colitis		No. of mice with dysplasia			
		Grade1	Grade2	Total	L- grade	H-grade	Adenoma
AOM/DSS	10	4(40%)	6(60%)	8(80%)	3(30%)	5(50%)	2(20%)
Emodin	9	4(44.4%)	5(55.6%)	7(77.8%)	6(66.7%)	1(11.1%)	0(0.0%)

L-grade, Low grade; H-grade, High grade; AOM/DSS, AOM/DSS treated mice; Emodin, AOM/DSS/Emodin treated mice.

From these data we conclude that Emodin suppresses inflammatory events and appearance of dysplastic lesion and adenoma early in the tumorigenic cascade.

### Emodin Inhibits LPS-Induced Pro-Inflammatory Gene Expression in RAW 264.7 Cells

Tissue resident macrophages are essential initiators of the inflammatory tissue response, including in colon cancer (35). As Emodin suppressed the acute inflammatory phase of AOM/DSS-induced colon carcinogenesis, we tested whether Emodin may suppress the acute activation of macrophages in response to an inflammatory stimulus. To this end we investigated the effect of Emodin on the expression of NO and TNFα in the macrophage cell line RAW 264.7 in response to LPS, a potent, natural pro-inflammatory macrophage agonist. No cytotoxicity was observed on RAW 264.7 cells exposed to 10, 20, and 40 μM Emodin, while decreased viability was seen with 60 μM (**Supplementary Figure S4A**). We next tested the response of RAW 264.7 cells to LPS in the absence or presence of Emodin (10, 20, and 40 μM). Emodin significantly and dose-dependently suppressed the enhanced production of NO (**Supplementary Figure S4B**) and TNFα (**Supplementary Figure S4C**) in RAW 264.7 cells stimulated for 24 h with LPS.

From this experiment we concluded that Emodin inhibits the acute inflammatory response of LPS-stimulated macrophages.

### Emodin Reduces Colon Cancer Cell Viability, Adhesion, and Migration

**Figure 5:**
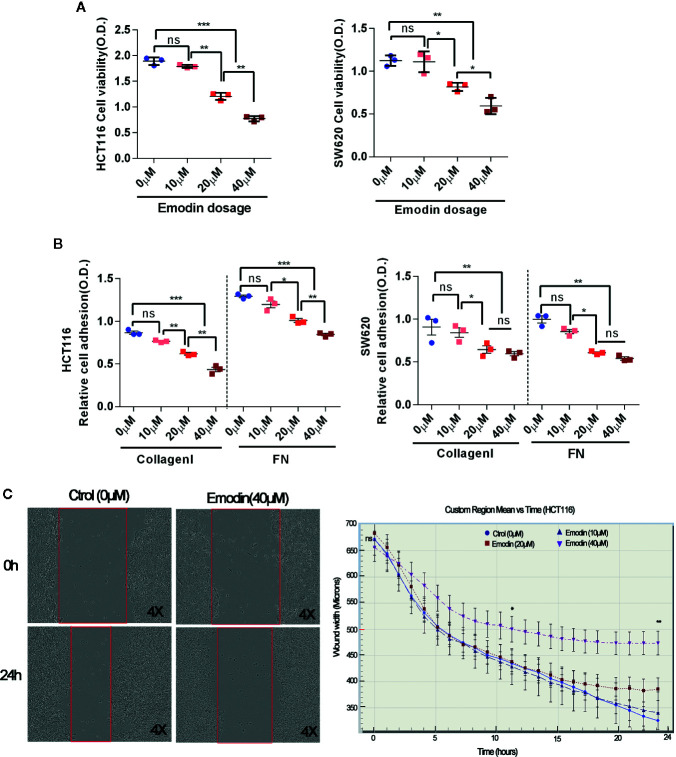
Emodin reduces colon cancer cell viability, adhesion, migration. **(A)** Viability of HCT116 and SW620 cells cultured in the absence or presence of 10, 20, 40 μM Emodin for 24 h. Viability was measured by MTT assay. **(B)** Adhesion on collage I or fibronectin (FN) of HCT116 and SW620 cell, pre-treated or not with Emodin (10, 20, 40 μM Emodin) for 24 h. Cell adhesion is assessed by crystal violet staining. **(C)** Migration of HCT116 cells treated or not with Emodin (40 μM) evaluated for 24 h in a scratch-wound healing assay. Micrographs show the actual wells, the graph show the plotted cell migration over time. Four to 6 wells for each group and experiments were repeated three times. *p < 0.05, **p < 0.01, ***p < 0.001, ns, not significant.

As Emodin reduced incidence, growth and invasion of AOM/DSS-induced carcinoma, we tested for direct effects of Emodin on viability, adhesion, and migration of colon cancer cell lines *in vitro*. As shown in **Figure 5A**, Emodin inhibited the viability of SW620 and HCT116 cells, two aggressive human CRC-derived cancer cell lines, in a dose-dependent manner (10, 20, and 40 μM). Emodin also dose-dependently inhibited adhesion of HCT116 and SW620 cells on fibronectin (FN) and collagen I (**Figure 5B**). To measure migration, we performed a scratch-wound migration assay, whereby the distance between the edges of an injured HCT116 monolayer (wound width) was measured hourly for 24 h. The results showed that Emodin (40 μM) decreased HCT116 cell migration starting 11 h after wounding (**Figure 5C**).

### Emodin Inhibits Colon Cancer Cell Growth and Fibroblasts-Induced Cancer Cell Invasion

**Figure 6:**
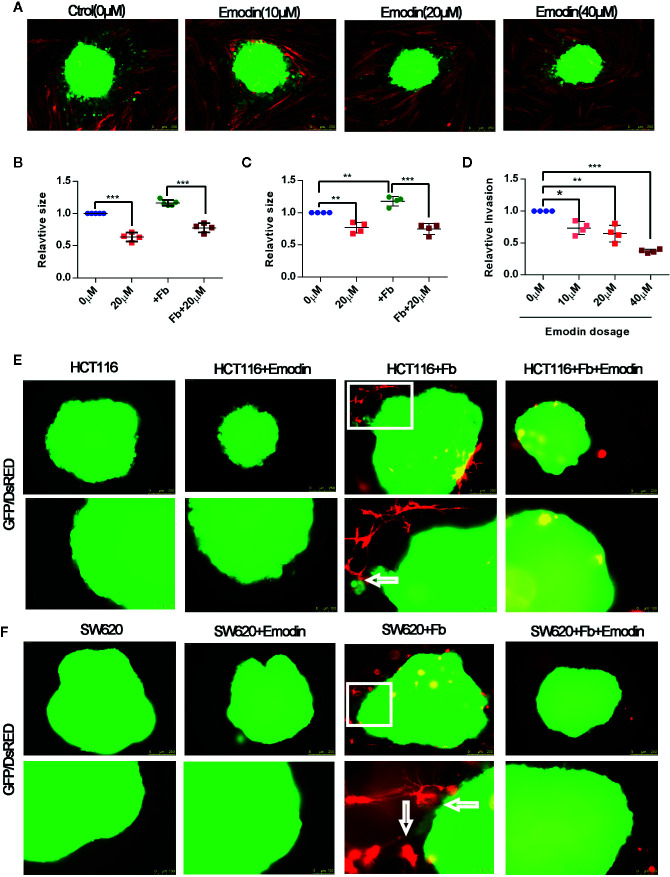
Emodin inhibits colon cancer cell growth and fibroblasts-induced cancer cell invasion. **(A–D)** 2D spheroids growth and invasion assay: **(A)** 2D spheroids invasion assay. GFP-HCT116 cell spheroids (green) were placed on a monolayer of DsRED-fibroblasts (red) in the absence or presence of 10, 20, 40 μM of Emodin for 3 days, as indicated. Representative images are shown; **(B)** relative size of HCT116 spheroids; **(C)** relative size of SW620 spheroids co-cultured in the absence or presence of fibroblasts ± Emodin (20 μM); **(D)** Relative quantification of invasion of HCT116 spheroids. No. of spheroids = 4 or 5 in each group. **(E, F)** 3D spheroids invasion assay. **(E)** HCT116 or **(F)** SW620 cells spheroids are co-cultured in Matrigel with or without DsRED-fibroblasts ± Emodin (20 μM) for 5 days. Representative images are shown and arrows indicate site of fibroblast-spheroid contacts with elongated tumor cells. No. of spheroids = 4 or 5 in each group. Fb, fibroblast. *p < 0.05, **p < 0.01, ***p < 0.001.

At week 14 after tumor initiation, we observed that Emodin reduced the presence of fibroblasts in the surrounding tumor microenvironment (**Figure 2G**). Cancer associated fibroblasts are critical determinants of tumor progression (33). We have previously reported that activated fibroblasts promote SW620 and HCT116 CRC cell growth and invasion (30). To test whether Emodin, may impinge on fibroblasts-promoted CRC cell growth and invasion we performed 2D and 3D tumor spheroid-fibroblasts co-culture assays. SW620 and HCT116 CRC cells were GFP tagged, while fibroblasts were DsRED tagged. The size of HCT116 and SW620 spheroid was decreased after Emodin treatment (20 μM) regardless of the presence or absence of fibroblasts (**Figures 6A–C**). Concomitantly, we monitored the effect of Emodin on tumor cell invasiveness in 2D conditions in the presence or absence of fibroblasts as described (30). Fibroblasts induced HCT116 cell invasion out of the spheroids, which was significantly inhibited by Emodin in a dose-dependent manner (**Figures 6A, D**).

Next, we performed the 3D invasion assay in which CRC cell spheroids were embedded in fibroblasts-supplemented Matrigel. Results, shown in **Figures 6E F**, demonstrated that fibroblasts (red) established contacts with tumor spheroids resulting in tumor cell elongation and invasion. In the presence of Emodin, however, there were no fibroblasts establishing contacts with the cancer cells, and no invading cancer cells were observed (**Figures 6E, F** **)**. In bright field images rounded fibroblasts were visible dispersed in the gel suggesting the Emodin impinged on their elongation/migration toward the spheroids (**Supplementary Figure S5**). Spheroids cultured alone did not develop signs of invasion, while Emodin treatment decrease the size of the tumor spheroids, consistent with the results shown in panel **Figure 6A**.

Taken together, these results demonstrate that Emodin treatment significantly inhibits the growth of human CRC cell line derived spheroid, and suppresses fibroblasts-induced CRC cell invasion in 2D and 3D conditions.

## Discussion

Colorectal cancer (CRC) is the third most common malignancy worldwide, and inflammation is an important promoter of CRC development (1, 2). Chronic inflammation plays a critical role in CRC development and CRC associated with IBD is more aggressive and difficult to treat (5). Inflammation affects every step of tumor development, including initiation, promotion, invasion and metastasis, and inhibition of inflammation was shown to have therapeutic benefits (3, 4). The Rhubarb plant is a natural medicine in China and Japan (36) with anti-inflammatory and anti-tumor activities (14). Emodin, one of the potent active compounds isolated from Rhubarb, was shown to exhibit anti-inflammatory effects in various diseases such as pancreatitis, arthritis, asthma, and atherosclerosis, by impinging on oncogenic inflammatory pathways and mediators, including NF-κB, TNFα, VEGF and chemokines (16). Emodin was reported to inhibit experimental tumors including of the colon (20). We started this study to investigate whether Emodin may inhibit the development and progression of colon cancer and unravel possible effects on the inflammatory response in the tumor microenvironment.

Here, using the AOM/DSS induced colitis-associated intestinal tumorigenesis model, we report that Emodin: i) decreased the incidence of epithelial hyperplasia and dysplasia at week 3, the incidence of intestinal carcinomas at week 5, and the incidence, size and invasiveness of intestinal carcinomas at weeks 14; ii) reduced CD11b^+^ and F4/80^+^ inflammatory cell infiltration and expression of mediators of inflammation (i.e. TNFα, COX-2, IL1α/β, IL6, NOS2, CCL2, CCL5, CXCL5) throughout the carcinogenesis process, while promoting recruitment of CD3^+^ T lymphocytes at 14 weeks; iii) inhibited the expression of pro-inflammatory mediators by LPS-stimulated RAW 264.7 macrophages *in vitro*; iv) reduced viability, adhesion, migration and fibroblasts-induced invasion of SW620 and HCT116 colon cancer cells *in vitro*.

Immune/inflammatory cell infiltrates are associated with CRC progression (37). Macrophages are the most abundant immune cells in the tumor microenvironment. They are derived from monocytic precursors in the blood and recruited to the tumor site by chemokines (38). Bader *et al.* found that macrophage depletion by clodronate administration during late-stage tumorigenesis in the AOM/DSS mouse model, reduced tumor growth (38, 39). We found that Emodin inhibited the recruitment of F4/80 positive macrophage in the colon tissue at the early and late stage of inflammation-induced CRC. In *in vitro* experiment, we found that Emodin suppresses the production of NO and TNFα in LPS activated RAW264.7. These data suggest that Emodin may reduce CRC carcinogenesis by suppressing inflammation early on in the process.

Inflammatory mediators play an important role in tumor progression and directly affect the tumor microenvironment. Expression of the inducible cyclooxygenase COX-2, is increased during inflammation and in many cancers, including CRC (40). COX-2 expression is increased during colorectal inflammation, at premalignant lesions and in CRC (41). Preclinical evidence indicates that COX-2 promotes CRC incidence and progression and selective COX-2 inhibition prevents these effects, suggesting that suppression of inflammation in general, and COX-2 inhibition in particular, may be a reasonable approach for the prevention and treatment of CRC (42, 43). Our results also show that at the early (week 5) or late (week 14) stages of tumorigenesis, COX-2 was strongly expressed in inflammatory and tumor cells, and Emodin significantly reduced its expression, especially in tumor cells. The changes in COX-2 mRNA paralleled changes in protein expression, suggesting that COX-2 regulation occurred at transcriptional level. Thus, Emodin appears to be a natural modulator of COX-2 in intestinal inflammation and colon cancer. Other inflammatory mediators involved in CRC, such as TNFα and NOS2, were also inhibited by Emodin during the progression from inflammation to carcinoma, through transcriptional regulation.

Myeloid-derived suppressor cells (MDSCs), a heterogeneous population composed of immature myeloid cells can accumulate in the blood and at tumor sites in cancer patients and in experimental cancer models, including in CRC. MDSCs inhibit both innate and adaptive immune responses and promote cancer-associated inflammation dampening anti-tumor immune responses (44). Li *et al.* investigated the role of MDSCs in neoplastic progression of AOM/DSS CRC mice, and flow cytometry analysis showed that AOM/DSS colonic tumors were more densely infiltrated with CD11b^+^Gr-1^+^ MDSCs compared to normal tissue (45). We observed that the number of MDSCs in peripheral blood decreased significantly in the AOM/DSS group, consistent with the possibility that they were recruited to the colon lesions. In accordance with these data, the staining for CD11b, a general marker of monocytes/macrophage, neutrophils and NK cells, was more abundant in tumors of the AOM/DSS group. Emodin promoted CD11b^+^ cells recruitment during acute inflammation, while it inhibited it in late tumorigenesis consistent with reversal of tumor-mediated immunosuppression.

Consistent with the latter observation, Emodin decreased the presence of CD3^+^ T cells during acute inflammation (week 5), while it stimulated their recruitment during late stage tumorigenesis (week 14). Various studies have indicated that tumor infiltrating CD3^+^ T lymphocytes have a prognostic value in CRC. High CD3^+^ cell infiltration at the invasive tumor margin correlates with a better disease-free survival and a better patient prognosis (46). Thus, Emodin, while suppressing myeloid-cell driven inflammation and MDSC recruitment, appears to restore T cell recruitment at late stage of tumorigenesis consistent with a reversal of immunosuppression.

Cancer-associated fibroblasts (CAF) are potent promoter of tumor growth, invasion and metastasis, including in CRC (33). Heichler *et al.* reported that STAT3 activation in CAFs promotes CRC development and the phosphorylation of STAT3 CAFs inversely correlated with patient survival (47). Sasaki *et al.* demonstrated that the CCL3-CCR5 chemokines axis mediated fibroblast accumulation was evident in colon, as well as leukocyte infiltration to promote colitis-associated carcinogenesis (48). We have previously shown that fibroblasts mediate CRC cell migration and invasion through FGFR-SRC signaling (30, 49). Emodin decreased the amount of CAF in AOM/DSS tumors in *in vivo* (**Figure 2**), suppressed fibroblasts-induced CRC cell invasion in the 2D and 3D assays *in vitro* (**Figure 6**), reduced fibroblasts content in the 3D assay *in vitro* (**Figure 6**) and reduced fibroblast viability under standard 2D culture conditions (data not shown). These observations suggest that CAF is an additional relevant cell population of the tumor microenvironment targeted by Emodin contributing to reducing tumor growth and progression.

Emodin, also inhibited survival, adhesion and migration of isolated CRC cells *in vitro*, consistent with direct effects on cancer cells as previously suggested (17–21).

## Data Availability Statement

The raw data supporting the conclusions of this article will be made available by the authors, without undue reservation.

## Ethics Statement

The animal study was reviewed and approved by Service de la sécurité alimentaire et des affaires vétérinaires SAAV Protection des animaux Impasse de la Colline 4, 1762 Givisiez.

## Author Contributions

Conceptualization: YZ, WP, L-kS, and CR. Investigation: YZ, WP, and SC. Methodology: YZ, WP, SC, JZ, MB, L-kS, and CR. Formal analysis: YZ, WP, MB, SC, JZ, L-kS, and CR. Resources: CR and L-kS. Funding acquisition: CR and L-kS. Supervision: SC, MB, L-kS, and CR. Writing—original draft, YZ, WP, L-kS, and CR. Writing—review and editing, YZ, MB, SC, JZ, WP, L-kS, and CR. All authors contributed to the article and approved the submitted version.

## Funding

This study was supported by Sino Swiss Science and Technology Cooperation (SSSTC) program (Grant: EG 07-032016 to CR and L-kS), Tianjin University of Traditional Chinese Medicine Foundation (to L-kS); Cancer Research Switzerland (KSF 4400-02-2018 to CR), the Swiss National Science Foundation (31003A_179248, to CR and 200021_184687 to GA).

## Conflict of Interest

The authors declare that the research was conducted in the absence of any commercial or financial relationships that could be construed as a potential conflict of interest.
