# Supplementary material for: Emodin Inhibits Inflammation, Carcinogenesis, and Cancer Progression in the AOM/DSS Model of Colitis-Associated Intestinal Tumorigenesis
Source: Front Oncol. 2021 Jan 8;10:564674. doi: 10.3389/fonc.2020.564674 (PMC7821392; doi:10.3389/fonc.2020.564674)
Supplement: Supplementary file 1 [file DataSheet_1.pdf]

Supplementary Material

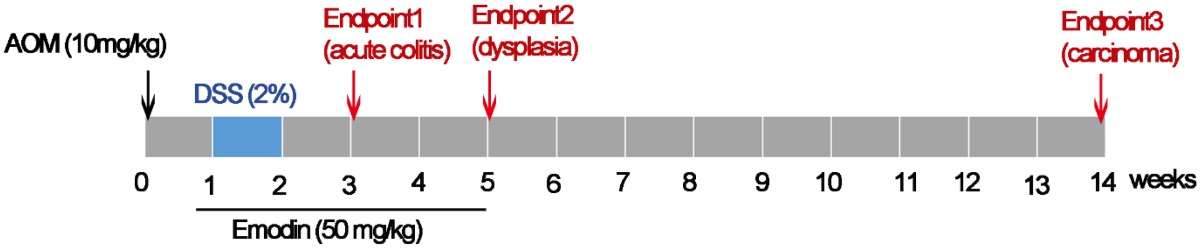

Supplementary Figure 1. Treatment protocol of the AOM/DSS model of colitis-associated intestinal tumorigenesis. Mice are injected intraperitoneally with azoxymethane (AOM, 10 mg/kg body weight) at day 0, followed after 1 week by one cycle of dextran sulfate sodium (DSS, 2% w/v, dissolved in drinking water) for 7 days. Emodin (50 mg/kg) or the vehicle solution (0.2% Tween80 and 0.5% Methyl cellulose in ddH_2O) was administrated by gavage, starting 2 days before DSS administration and continued for 4 weeks. Mice were sacrificed at three different endpoints: week 3 when DSS induced acute colitis; week 5 when colonic crypts showed dysplasia and initial carcinoma; week 14 when 100% incidence of carcinomas occurred in the AOM/DSS group.

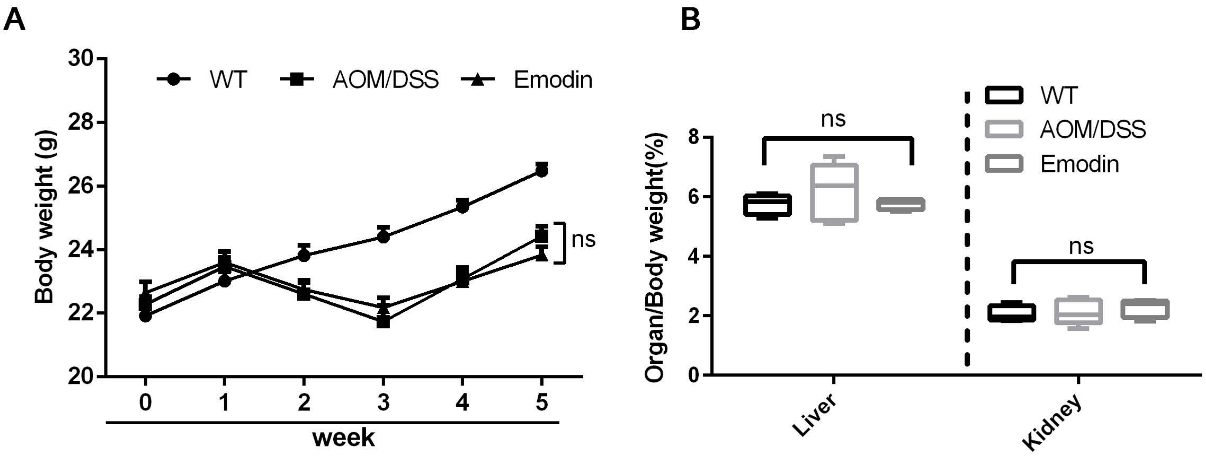

Supplementary Figure 2. Emodin exerts no toxicity under the chosen experimental dosage. (A) Body weight of experimental groups from week 0 to 5. **(B)** The liver and kidney index (tissue weight/body weight in %) of each group at week 5. n=10 in WT; n=15 in group AOM/DSS and Emodin, respectively; WT: tumor free mice; AOM/DSS: AOM/DSS treated mice; Emodin: AOM/DSS/Emodin treated mice. ns: not significant.

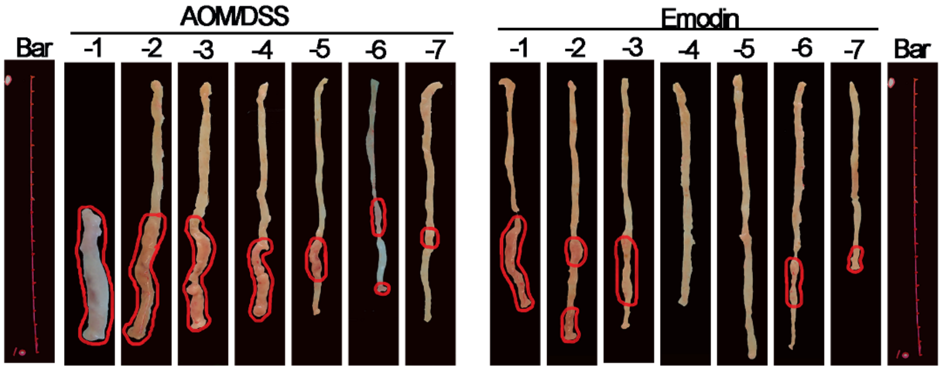

Supplementary Figure 3. Macroscopic views of the colons of mice. Mice were treated first with AOM/DSS to induce tumorigenesis and subsequently with vehicle only (AOM/DSS) or Emodin (See Supplementary Figure 1). Emodin-treated mice have visibly fewer and smaller tumors (circled in red).

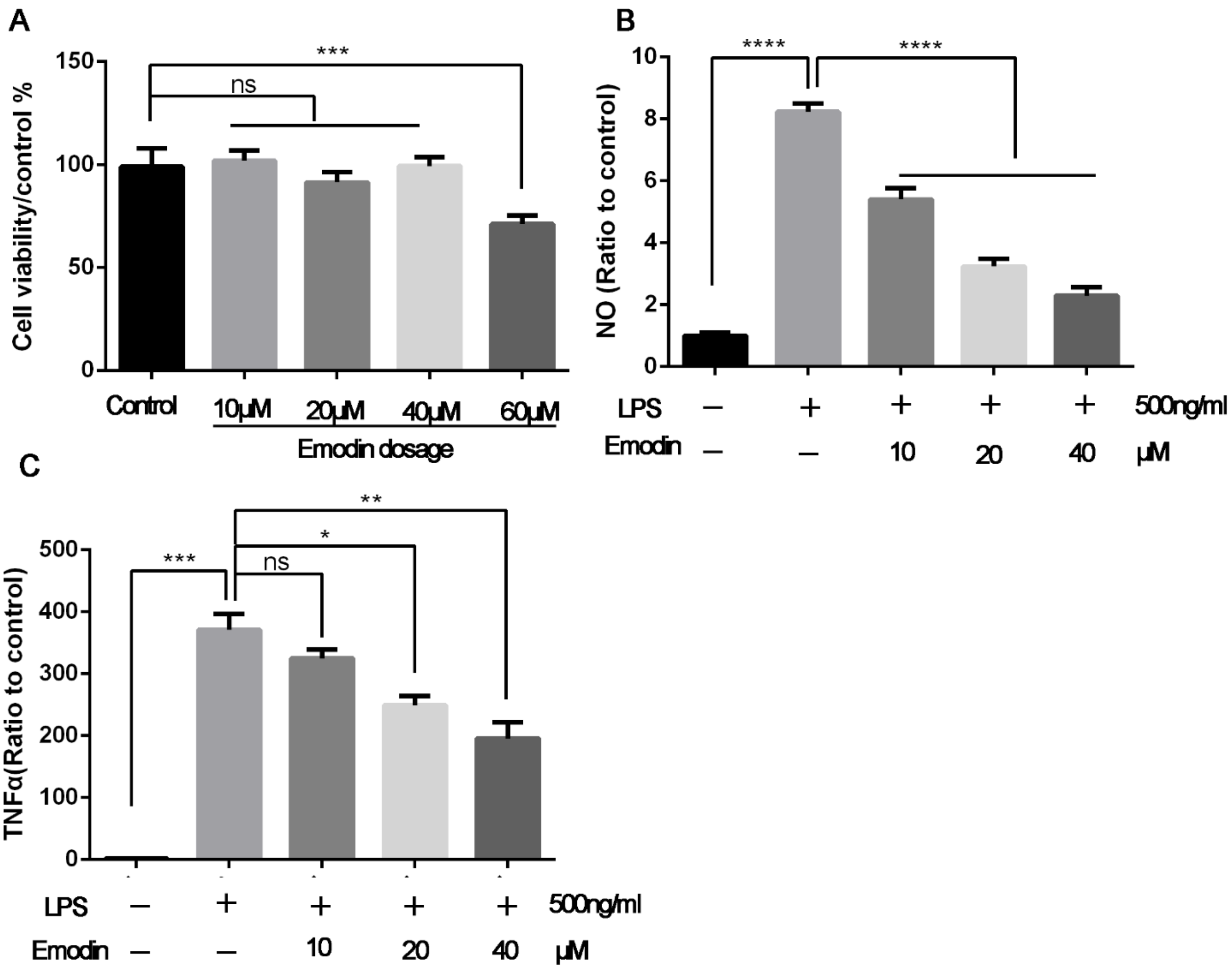

Supplementary Figure 4. Emodin inhibits the acute inflammatory responses of LPS-stimulated RAW 264.7 macrophage line. **(A)** Viability of RAW 264.7 cells cultured in the absence or presence of 10, 20, 40, 60 μM Emodin for 24 hours. Viability was measured by MTT assay. **(B)** Production of NO (measured by Griess reagent) in the culture medium of RAW 264.7 cell stimulated with LPS for 24 hours. **(C)** Production of TNFα (measured by ELISA) in the culture medium of RAW 264.7 stimulated with LPS for 24 hours. Experiments were repeated three times. *p < 0.05, **p < 0.01,***p < 0.001, ****p < 0.0001, ns: not significant.

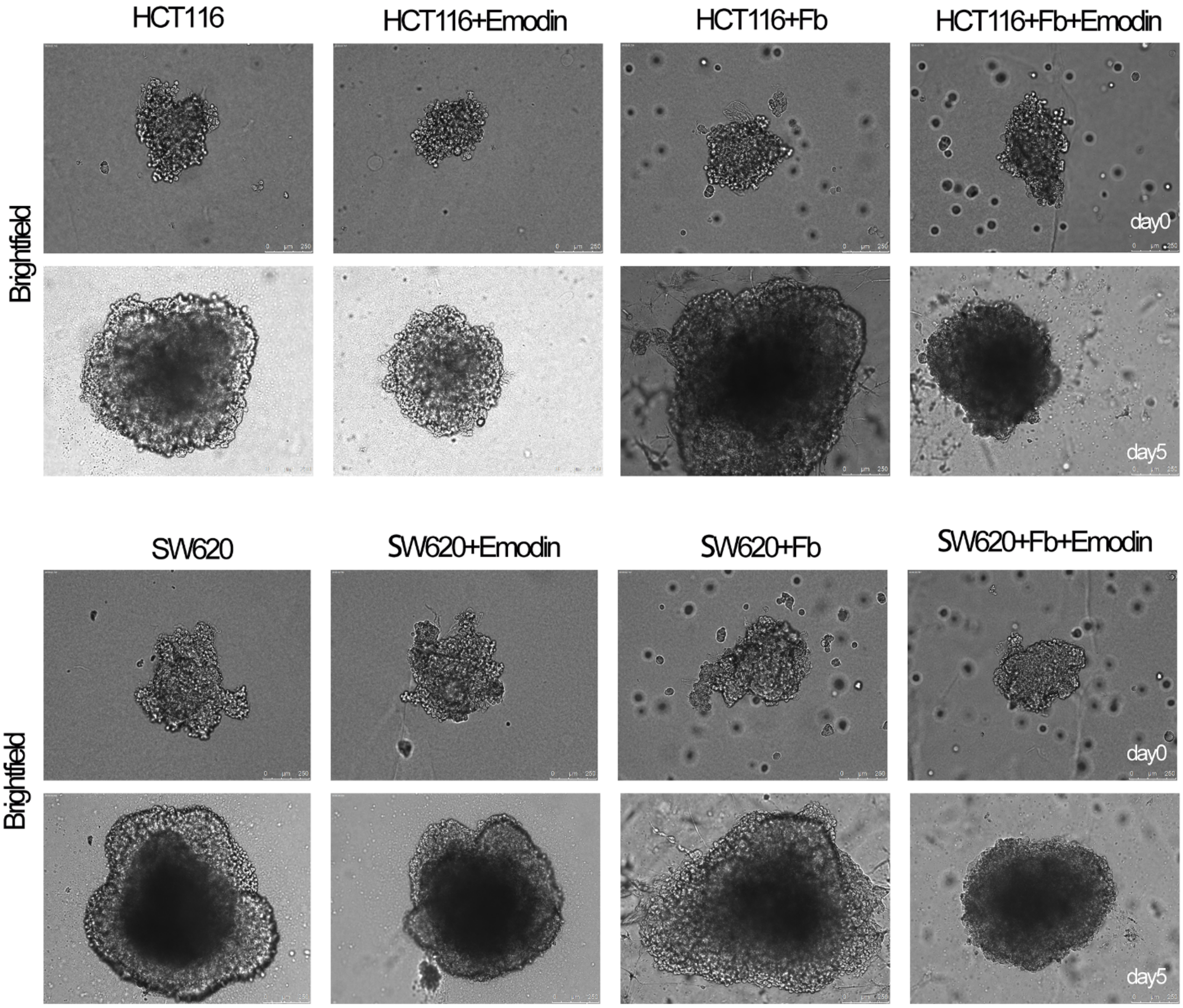

Supplementary Figure 5. Emodin inhibits colon cancer cell growth and fibroblasts-induced cancer cell invasion. Brightfield images of the 3D spheroids invasion assay shown in main Figure 6: HCT116 or SW620 cells spheroids are co-cultured in Matrigel with or without DsRED-fibroblasts ± Emodin (20 μM). Representative images for spheroids are shown at day 0 and day 5. Emodin prevents fibroblasts from elongating and migrating toward the tumor spheroids. No. of spheroids: 4-5 in each group.
